# Pautas éticas para la reanimación cardiopulmonar en el contexto de la pandemia de COVID-19 en Colombia

**DOI:** 10.7705/biomedica.5762

**Published:** 2020-11-15

**Authors:** Eduardo A. Rueda, Edilma Suárez, Fritz E. Gempeler, Lilian Torregrosa, Andrea Caballero, Diana Bernal, Nora Badoui

**Affiliations:** 1 International Bioethics Committee - UNESCO, Red Latinoamericana y del Caribe de Educación en Bioética; Facultad de Derecho y Ciencias Políticas, Universidad Nacional de Colombia, Bogotá, D.C., Colombia Universidad Nacional de Colombia Facultad de Derecho y Ciencias Políticas Universidad Nacional de Colombia BogotáD.C Colombia; 2 Facultad de Enfermería, Pontificia Universidad Javeriana, Bogotá, D.C., Colombia Pontificia Universidad Javeriana Facultad de Enfermería Pontificia Universidad Javeriana BogotáD.C Colombia; 3 Facultad de Medicina, Pontificia Universidad Javeriana, Bogotá, D.C., Colombia Pontificia Universidad Javeriana Facultad de Medicina Pontificia Universidad Javeriana BogotáD.C Colombia; 4 Servicio de Ética, Hospital Universitario de San Ignacio, Bogotá, D.C., Colombia Hospital Universitario de San Ignacio BogotáD.C Colombia; 5 Asociación Colombiana de Cirugía, Bogotá, D.C., Colombia Asociación Colombiana de Cirugía BogotáD.C Colombia; 6 Departamento de Cirugía, Hospital Universitario San Ignacio, Bogotá, D.C., Colombia Departamento de Cirugía Hospital Universitario San Ignacio BogotáD.C Colombia; 7 Clínica Campo Abierto, EPS Sanitas, Bogotá, D.C., Colombia EPS Sanitas BogotáD.C Colombia; 8 Facultad de Jurisprudencia, Universidad del Rosario, Bogotá, D.C., Colombia Universidad del Rosario Facultad de Jurisprudencia Universidad del Rosario BogotáD.C Colombia; 9 Consejo Nacional de Bioética de Colombia, Bogotá, D.C., Colombia Consejo Nacional de Bioética de Colombia BogotáD.C Colombia; 10 Facultad de Medicina, Universidad Nacional de Colombia, Bogotá, D.C., Colombia Universidad Nacional de Colombia Universidad Nacional de Colombia BogotáD.C Colombia

**Keywords:** infecciones por coronavirus, reanimación cardiopulmonar, códigos de ética, guía de práctica clínica, Coronavirus infections, cardiopulmonary resuscitation, codes of ethics, practice guideline

## Abstract

La pandemia de COVID-19 se ha asociado con un incremento en el número de casos de paro cardiorrespiratorio y con ello han aumentado las inquietudes éticas en torno a la exigencia de la reanimación cardiopulmonar, así como sobre las condiciones para realizarla. El riesgo de contagio por aerosoles y las incertidumbres clínicas sobre la eficacia, las potenciales secuelas y las circunstancias que podrían justificar la limitación del procedimiento durante la pandemia, han multiplicado las dudas éticas sobre cómo proceder en estos casos. Con base en fundamentos éticos y jurídicos, en el presente artículo se ofrece una guía práctica sobre cómo proceder en los casos de paro cardiopulmonar en el contexto de la pandemia. Los criterios de justicia, beneficio, no daño, respeto a la autonomía, precaución, integridad y transparencia, se presentan de forma organizada y práctica para la adopción de decisiones en materia de reanimación cardiopulmonar.

## Abstract (en)

The pandemic caused by COVID19 is associated with an increase in the number of cases of cardiorespiratory arrest, which has resulted in ethical concerns regarding the enforceability of cardiopulmonary resuscitation, as well as the conditions to carry it out. The risk of aerosol transmission and the clinical uncertainties about the efficacy, the potential sequelae, and the circumstances that could justify limiting this procedure during the pandemic have multiplied the ethical doubts on how to proceed in these cases. Based on ethical and legal grounds, this paper offers a practical guide on how to proceed in the clinical setting in cases of cardiopulmonary arrest during the pandemic. The criteria of justice, benefit, no harm, respect for autonomy, precaution, integrity, and transparency are asserted in an organized and practical framework for decision-making regarding cardiopulmonary resuscitation.

El incremento progresivo de casos de COVID-19 en Colombia conlleva el aumento paralelo en el número de casos de personas con paro cardiopulmonar que llegan a los servicios de urgencias o que ocurren durante las hospitalizaciones. Esta situación, sumada a la carencia de suficientes y adecuados elementos de protección personal en muchos de los hospitales y clínicas del país, suscita inquietudes en los equipos clínicos en torno a los criterios éticos y técnicos para decidir si se realizan o no maniobras de reanimación cardiopulmonar en casos específicos. Estas inquietudes responden al riesgo de contagio que los aerosoles y gotas producidas por los pacientes en paro durante el procedimiento de la reanimación cardiopulmonar representan para el personal clínico y para otros pacientes ^1^. En este contexto surgen varias preguntas: ¿es éticamente aceptable que, aunque no exista certeza científica sobre la capacidad de los aerosoles de producir el contagio de COVID 19, se modifiquen las pautas ya establecidas para las maniobras de la reanimación cardiopulmonar? ¿Es éticamente obligatorio hacer la reanimación cardiopulmonar a todo paciente con paro cardiopulmonar en el marco de la pandemia de COVID-19? ¿Es éticamente aceptable que los equipos clínicos se nieguen a realizar maniobras de reanimación cardiopulmonar si no cuentan con suficientes y adecuados elementos de protección personal?

La Organización Mundial de Salud (OMS) ha difundido evidencia sobre la elevada producción de aerosoles durante las maniobras de la reanimación cardiopulmonar, especialmente en la intubación ^2^. Se desconoce aún si estos siempre portan virus o si su potencial para producir contagios aumenta o disminuye dependiendo del clima, la presión atmosférica o la ventilación local ^3^^,^^4^. A pesar de estas incertidumbres, en los casos en que la infección de COVID-19 amenace la vida de quienes la padecen, se justifica toda medida que proteja al personal clínico contra el riesgo de contagio por el virus, como bien lo ha señalado la OMS.

Las dudas en cuanto a la pertinencia de las maniobras de la reanimación cardiopulmonar en el marco de la pandemia han revivido, por otra parte, discusiones en torno a las circunstancias clínicas en las que se justificaría realizar o no dichas maniobras. Efectivamente, en el contexto de estos debates se han determinado diversas circunstancias en las que la reanimación cardiopulmonar no se justificaría clínicamente, y se han discutido las formas de informar a los pacientes y a sus familias y comunidades, sobre la eficacia clínica y los resultados asociados con el procedimiento.

Con base en principios éticos y constitucionales sólidamente establecidos, en este artículo se presentan pautas y recomendaciones para asegurar una práctica éticamente rigurosa de la reanimación cardiopulmonar en el marco de la pandemia de COVID-19.

## Fundamentos éticos y jurídicos

Las posibles respuestas a las preguntas éticas que se plantean sobre la reanimación cardiopulmonar en el marco de la pandemia de COVID-19 deben ajustarse a los siguientes principios ampliamente reconocidos en las sociedades democráticas.

### Principio de autonomía

Este principio obliga a acatar la expresión de voluntad de los pacientes en relación con los cursos de acción que, en el ámbito clínico, les proponga el equipo de salud ^5^. En el caso de la reanimación cardiopulmonar, este principio indica que esta es admisible solo si no se opone a la voluntad manifiesta del paciente, expresada mediante un documento de voluntad anticipada. Los pacientes pueden tomar decisiones bien sopesadas en esta materia solo si se les da información oportuna, suficiente y clara.

### Principio de beneficio y lex artis

Este principio exige que la atención médica esté justificada por el potencial beneficio del paciente. En el marco de las decisiones sobre la reanimación cardiopulmonar, este principio indica que estas se justifican únicamente cuando la reanimación cardiopulmonar suponga un beneficio real para el paciente según la *lex artis*^6^.

### Principio de no daño y respeto por el derecho a la integridad del paciente

Este principio ordena que a toda persona se le proteja de cualquier daño que pueda sobrevenir por causa de la atención médica. En el contexto que nos ocupa, este principio obliga a hacer la reanimación cardiopulmonar solo en aquellos casos en que esta no constituya una futilidad o una forma de encarnizamiento terapéutico ^6^^,^^7^. Su aplicación debe garantizar la protección del derecho a la integridad del paciente, que es conexo con el derecho a la salud.

### Principio de precaución

Este principio obliga a hacer la reanimación cardiopulmonar solo en aquellos casos en que no se exponga a los pacientes y al personal clínico y administrativo de las instituciones de salud a graves riesgos de salud ^5^^-^^9^. En el marco de este principio, deben garantizarse todas las medidas y equipos de protección personal al personal de salud antes de cualquier procedimiento que suponga riesgo de contagio para ellos, incluso cuando no exista certeza completa sobre la magnitud y el alcance de dicho riesgo (principio de precaución) ^5^^,^^8^^,^^10^.

### Principio de justicia

Este principio obliga a garantizar a las personas las mejores condiciones de cuidado clínico disponibles, sin discriminación alguna. Cuando los recursos son limitados este principio implica priorizar a quienes los recibirán considerando el mayor potencial de beneficio clínico a corto y largo plazo y la equidad de oportunidades entre los ciudadanos para vivir un ciclo de vida completo. En el contexto que nos ocupa, este principio justifica limitar la reanimación cardiopulmonar cuando de su práctica se derive la demanda de recursos escasos, como camas en las unidades de cuidados intensivos, respiradores, etc., que deberían ser asignados a otras personas según los mencionados principios ^11^.

### Principio de integridad

Este principio establece la obligación de proveer los cuidados hospitalarios con base en los más altos estándares de profesionalismo, procurando siempre el mejor cuidado posible para los pacientes ^9^. En el caso que nos ocupa, este principio obliga a disponer de procedimientos y grupos adecuadamente entrenados (equipos de código azul o respuesta rápida) para una debida reanimación cardiopulmonar.

### Principio de transparencia

Este principio exige que los criterios para la toma de decisiones sobre la reanimación cardiopulmonar sean pública y abiertamente conocidos por todos los involucrados a lo largo del proceso de atención hospitalaria, a saber, el equipo clínico, los pacientes, los familiares y las autoridades de salud ^7^^,^^8^.

### Comunicación, consentimiento y equipos de respuesta rápida

Como cualquier procedimiento hospitalario, la reanimación cardiopulmonar tiene sus indicaciones específicas. La eficacia clínica de ella, según lo reporta la *American Heart Association*^12^, es muy baja. En los casos de paro cardiopulmonar dentro de los hospitales la tasa general de éxito, definida como la supervivencia de los pacientes al salir del hospital, oscila entre el 6,5 (en los casos de paro cardíaco no presenciado) y el 24 % (en el paro presenciado) ^12^^,^^13^. Por otra parte, en los estudios se ha evidenciado que solo entre el 1 y el 3 % de los pacientes con paro cardiopulmonar que reciben reanimación cardiopulmonar alcanza un nivel cognitivo cercano al que tenía antes ^14^^,^^15^. En el marco de la pandemia de COVID-19, la eficacia clínica de la reanimación cardiopulmonar podría ser aún más baja. En el estudio de Shao, *et al.,* en Wuhan, solo el 13,2 % de los pacientes con COVID-19 recuperaron la actividad cardíaca espontánea; el 2,9 % sobrevivió un mes, y solo un paciente de 136 sobrevivió sin mayores secuelas neurológicas ^16^. Además, como lo demuestran las estadísticas, en estos pacientes la mayoría de los paros no se asoció con la fibrilación ventricular sino con los ritmos no desfibrilables, que son de peor pronóstico ^7^.

Sin embargo, los pacientes y la comunidad no suelen saber de la poca eficacia clínica de la reanimación cardiopulmonar y la alta probabilidad de secuelas neurocognitivas en los supervivientes. A menudo, las ideas del público sobre la eficacia de la reanimación cardiopulmonar provienen de las películas, en las que los porcentajes de eficacia clínica sin secuelas superan el 70 %, o de información inexacta difundida por los medios o las redes sociales ^17^. Incluso entre los médicos, el pleno conocimiento sobre los niveles de eficacia clínica y las potenciales secuelas asociadas con la reanimación cardiopulmonar es poco común.

En este contexto, y con base en las obligaciones indicadas por los principios de autonomía, beneficio y no daño, es necesario que los pacientes cuenten con información clara sobre la eficacia clínica de la reanimación cardiopulmonar, las secuelas que puede tener y las circunstancias en las que se justificaría abstenerse de realizar el procedimiento ^7^. Solo así estarían en condiciones de decidir anticipadamente su voluntad ante estas situaciones ^18^^,^^19^. La educación pública sobre estas materias resulta, por lo tanto, crucial. La tarea de informar debe estar a cargo de los equipos tratantes con el apoyo de los comités clínicos de priorización ^11^ responsables de la asignación de recursos escasos, o de los comités de ética asistencial.

La recomendación internacional estándar frente a la reanimación cardiopulmonar es la conformación de equipos de código azul o de respuesta rápida, los cuales deben incluir, idealmente, un anestesiólogo, un intensivista, un internista y una o dos enfermeras jefes, o una enfermera jefe y una auxiliar de enfermería, todos ellos con amplia experiencia ^18^. En el marco de la pandemia de COVID-19 resulta especialmente crucial seguir esta recomendación sobre el número de integrantes y las características de los equipos de respuesta rápida para asegurar la calidad del procedimiento de la reanimación cardiopulmonar, pues se sabe que los equipos de protección personal aumentan la carga física y los niveles de cansancio del personal clínico.

## Guía práctica para la reanimación cardiopulmonar

Los principios éticos explicados en el segundo apartado se traducen en mandatos específicos frente a las decisiones en torno a la reanimación cardiopulmonar y deben acatarse para el manejo clínico de todos los pacientes, es decir, tanto los afectados por la COVID-19 como los no infectados. Según estos principios, la reanimación cardiopulmonar es éticamente aceptable mientras:

- no menoscabe las pautas de asignación justa de recursos sanitarios escasos,
- no contravenga las decisiones autónomas de los pacientes,
- no se lleve a cabo sin que los equipos de respuesta rápida cuenten con todos los equipos de protección personal necesarios para protegerse del contagio por el virus, y
- no suponga un modo de encarnizamiento terapéutico. Estos mandatos deberán aplicarse en estricto orden.

### a. No actuar de forma injusta

Cuando haya escasez de respiradores no deben realizarse maniobras de reanimación cardiopulmonar en aquellos pacientes que no hayan sido priorizados con base en las reglas de asignación éticamente justificadas, pues eventualmente requerirían de respiradores que pueden no estar disponibles ^11^. A los pacientes que no tengan prioridad se les deben garantizar todas las medidas de cuidado paliativo y confort físico, emocional y espiritual que requieran durante el final de su vida ^6^.

### b. Confirmación de la voluntad anticipada

A todo paciente que ingrese al hospital se le debe preguntar si cuenta con una declaración escrita de voluntad anticipada sobre la reanimación cardiopulmonar. Si ese es el caso, debe dejarse constancia de ella en la historia clínica ^5^^,^^19^. Si dicha declaración no se ha hecho, tras confirmar que el paciente no quedará exceptuado de la asignación de respiradores en caso de ser escasos y después de informarle sobre la eficacia y las secuelas potenciales, se le debe preguntar si, llegado el caso, aceptaría que se le practiquen maniobras de reanimación cardiopulmonar. Su decisión debe quedar consignada en la historia clínica, con su firma y la de un testigo. Si los pacientes están en situación de incompetencia, se les debe preguntar a sus familias si el paciente ha dejado por escrito su voluntad anticipada sobre la reanimación cardiopulmonar. Si no se cuenta con ella, se presumirá que el paciente consiente con el procedimiento de reanimación cardiopulmonar siempre que no existan razones para limitarlo ^5^^,^^19^. Esta misma presunción aplicará a todo paciente que ingrese al hospital en paro cardiopulmonar y que no disponga de una declaración de voluntad anticipada escrita. Se trata, así, de garantizar que no se practique la reanimación cardiopulmonar de un modo que deshonre el principio de dignidad y autonomía de los pacientes.

### c. Garantizar la protección adecuada del equipo clínico

Antes de llevar a cabo maniobras de reanimación cardiopulmonar es mandatorio que el equipo a cargo cuente con los elementos de protección personal necesarios para realizarlas bajo condiciones adecuadas de bioseguridad ^1^^,^^6^^,^^9^. Si no se dispone de estos, llevar a cabo maniobras de reanimación cardiopulmonar resultaría pugnante con el principio de no daño y los derechos a la vida y a la integridad física del equipo clínico y de otros pacientes, principios estos conexos con el de este apartado ^10^^,^^18^. Por las mismas razones, se impone restringir y limitar estrictamente al acceso del equipo de respuesta rápida a la zona destinada a la reanimación cardiopulmonar ^5^^,^^7^^,^^8^, así como garantizar adecuados sistemas de respiración asistida en dichas áreas.

### d. No incurrir en el encarnizamiento terapéutico

Por último, es fundamental que no se realice la reanimación cardiopulmonar cuando entrañe un modo de encarnizamiento terapéutico, es decir, cuando resulte fútil (incluso si ello contraviene la voluntad de la familia) ^6^, lo cual sucedería en los siguientes casos de paro cardiopulmonar.

- Cuando los pacientes tengan comorbilidades asociadas con una supervivencia menor a un año o en situaciones de grave fragilidad. Para determinar los casos de grave fragilidad puede emplearse la *Clinical Frailty Scale* (puntuación igual o mayor de 6) ^18^.
- Si el paciente con COVID-19 se encuentra en una unidad de cuidados intensivos en proceso de deterioro por incrementos de los parámetros contemplados en la escala de *Sequential Organ Failure Assessment* (SOFA) o en la *Pediatric Logistic Organ Dysfunction-2* (PELOD2).
- Si el paciente es mayor de 85 años.
- Si el paciente llega en paro cardiopulmonar, se le hayan hecho maniobras previas de reanimación cardiopulmonar o no, y tras más de 20 minutos de haberse presentado el paro.
- Si no se dispone de los equipos de respiración asistida y cuidado intensivo requeridos para darle continuidad al tratamiento, es decir, lo que se conoce como "cadena de supervivencia"

Con el fin de evitar la futilidad del procedimiento, varios autores recomiendan también que, cuando se hayan completado 20 minutos de maniobras de reanimación cardiopulmonar o cinco desfibrilaciones sin resultado, se detenga el procedimiento ^18^.

## Elementos de protección personal para el equipo de respuesta rápida

Debido a que en el momento de la reanimación cardiopulmonar se generan aerosoles, es necesario que el equipo de reanimación cuente con los equipos de protección personal adecuados para así reducir el riesgo de exposición ^4^^,^^6^^,^^8^. Estos equipos son diferentes a los usados en la atención rutinaria (tapabocas convencionales), tal como lo establecen los lineamientos del Ministerio de Salud y Protección Social y el Consenso ACIN-IETS de mayo de 2020 ^20^, además, siempre son de uso individual y por ningún motivo podrán compartirse ^2^.

Los decretos 488 ^21^ y 500 ^22^ de 2020 de la Presidencia de la República han establecido, por otra parte, que es obligación de las administradoras de riesgos laborales (ARL) suministrar estos equipos de forma correcta, suficiente y oportuna. Corresponderá al empleador exigir a las ARL, mediante las medidas pertinentes, el suministro pleno de los equipos de protección personal al equipo de salud.

## Recomendaciones finales

En el marco de la pandemia de COVID-19 es necesario adoptar pautas éticas sólidas para orientar en cada caso la decisión de recurrir a las maniobras de reanimación cardiopulmonar o no. En este documento el propósito ha sido presentarlas de manera sistemática. Las situaciones específicas y la disponibilidad de recursos en cada lugar obligan, sin embargo, a que estas pautas sean discutidas por los equipos clínicos a cargo de las maniobras de ella y adaptadas a su realidad particular. Este proceso de apropiación habrá de acompañarse de planes locales que incluyan un completo alistamiento de los equipos de respuesta rápida y la delimitación de zonas confinadas para la reanimación cardiopulmonar en los servicios de urgencias, así como la actualización de los equipos tratantes en las buenas prácticas de comunicación con los pacientes y familiares, la implementación de programas de información a la comunidad en materia de reanimación cardiopulmonar y, especialmente, la provisión suficiente y adecuada de elementos de protección personal a los equipos clínicos a cargo de los pacientes ^7^^,^^8^.

Como se ha indicado, existen razones éticas sólidas para condicionar la reanimación cardiopulmonar a la disponibilidad suficiente y adecuada de estos elementos para los equipos de respuesta rápida en todos los hospitales públicos y privados del país. Esperamos que esta compilación contribuya a que las autoridades sanitarias cobren plena conciencia de la obligación ética de dar la protección debida a todo el personal clínico hospitalario.
